# Role of the AIM2 Inflammasome in Cancer: Potential Therapeutic Strategies

**DOI:** 10.3390/biomedicines13020395

**Published:** 2025-02-06

**Authors:** Chiara Colarusso, Michela Terlizzi, Simone Di Caprio, Anna Falanga, Emmanuel D’Andria, Roberta d’Emmanuele di Villa Bianca, Rosalinda Sorrentino

**Affiliations:** 1 Department of Pharmacy (DIFARMA), University of Salerno, 84084 Fisciano, SA, Italy; ccolarusso@unisa.it (C.C.); mterlizzi@unisa.it (M.T.); sdicaprio@unisa.it (S.D.C.); afalanga@unisa.it (A.F.); emmdandria@unisa.it (E.D.); 2 Department of Pharmacy, University of Naples Federico II, 80131 Naples, NA, Italy; roberta.demmanueledivillabianca@unina.it

**Keywords:** AIM2, inflammasome, single-stranded DNA, ssDNA, cancer, natural and synthetic AIM2-targeted inhibitors, AIM2-based gene therapy, therapeutic strategies

## Abstract

Absent in melanoma 2 (AIM2) is a member of the innate immune sensors that recognizes cytosolic nucleic acids, leading to inflammasome assembly. In recent years, several studies in the oncology field have highlighted the presence of cytoplasmic double-stranded DNA (dsDNA) following necrosis and/or genomic instability, which is typical of malignant transformation. The recognition of dsDNA by the AIM2 inflammasome either in cancer cells or in immune cells can further exacerbate inflammatory processes on the basis of cancer progression. In this context, the role of AIM2 in cancer is still controversial in that some authors assume that AIM2 activation has pro-tumor activities, while others define it as anti-tumor. This discrepancy may be due to the nature of the cells where AIM2 is expressed or the histology of the tumor. This review aims to provide an overview of the controversial role of AIM2 in cancer, taking into consideration the pharmacological tools currently available to modulate AIM2 activity in cancer.

## 1. Introduction

**Figure 1:**
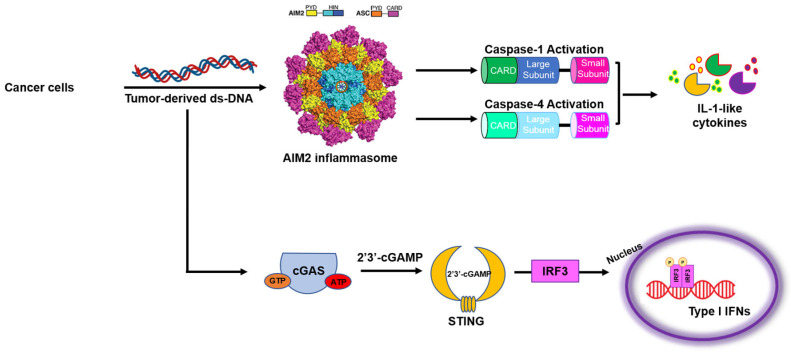
Tumor-derived double-stranded DNA (dsDNA), which can be derived by genomic instability, activates AIM2 inflammasome and cGAS-STING signaling pathway. The AIM2 receptor consists of an N-terminal PYD, which is responsible for the recruitment of the adaptor protein apoptosis-associated speck-like protein containing a CARD (caspase recruitment domain) (ASC), and a C-terminal HIN domain, which is responsible for the recognition of and binding with the DNA. After the recognition of dsDNA in the cytoplasm, AIM2 binds to ASC and protease caspase-1 and/or caspase-4 to form the AIM2 inflammasome intracellular multiprotein complex, leading to the release of active IL-1-like cytokines. Tumor-derived dsDNA can also be sensed by the cyclic GMP-AMP synthase (cGAS). Upon its activation, cGAS produces cyclic 2′3′GMP-AMP (2′3′cGAMP), which acts as a second messenger to activate stimulator of interferon genes (STING). STING activation leads to the recruitment of the interferon regulatory factor 3 (IRF3), a transcription factor, which drives the expression of type I IFN.

The detection of pathogen- or danger-associated molecular patterns (PAMPs or DAMPs) by host cells is a key strategy adopted by the innate immune system to guarantee physiological homeostasis. In this scenario, a pivotal role is played by pattern recognition receptors (PRRs) which are mainly expressed by immune cells and are able to trigger inflammatory events in response to endogenous (i.e., adenosine triphosphate (ATP), monosodium urate (MSU), cholesterol crystals, and β amyloid protein) and exogenous (i.e., pathogens like bacteria, fungi and viruses, asbestos, particulate matter, and UV radiation) insults. An alteration in PAMP and DAMP recognition/detection could contribute to the establishment of inflammatory/autoimmune diseases or tumorigenesis [1]. Among the intracellular DAMPs, endocytosed DNA derived from dying cells or damaged tissues, as well as self-DNA leaking from either mitochondria or nuclei, can activate an innate immune response that allows the humoral and cell-mediated adaptive immunity to exert its activity in the inflamed tissue [2]. In recent years, several immunological and oncological studies have focused on the role of genomic instability and/or the leakage of DNA as a key step for malignant transformation [1,3]. Particularly, self-DNA and cytoplasmic double-stranded DNA (dsDNA) released by cancer cells, byproducts of genomic instability, were reported to trigger the cytosolic absent in melanoma 2 (AIM2) (Figure 1) promoting tumor growth [4].

### AIM2 Inflammasome

The AIM2 receptor belongs to the Pyrin and hematopoietic interferon-inducible nuclear (HIN) domain (PYHIN) family (pyrin + HIN). It consists of an N-terminal PYD, which is responsible for the recruitment of the adaptor protein apoptosis-associated speck-like protein containing a CARD (caspase recruitment domain) (ASC), and a C-terminal HIN domain, which is responsible for the recognition of cytosolic DNA [5] (Figure 1). AIM2 is constitutively expressed in hematopoietic cells and is directly activated by dsDNA [6] derived by both Listeria- and Francisella-infected or virus-infected (i.e., Cytomegalovirus and Vaccinia) cells, as well as by endogenous DNA released during cell damage [7]. The research on AIM2 function and operating modes have revealed that the AIM2-DNA binding affinity depends on the DNA length. AIM2 has a higher affinity for dsDNA longer than 70–280 bp [8], but it can also sense DNA-RNA hybrids [9] and viral RNA [10]. It was suggested that the activation of dsDNA-induced AIM2 activation is associated with both a hydrophobic and electrostatic interaction between the backbone of the DNA (negatively charged) and the HIN domain (which contains positively charged amino acid residues) which drives the assembly of AIM2 molecules for inflammasome formation [10].

**Figure 2:**
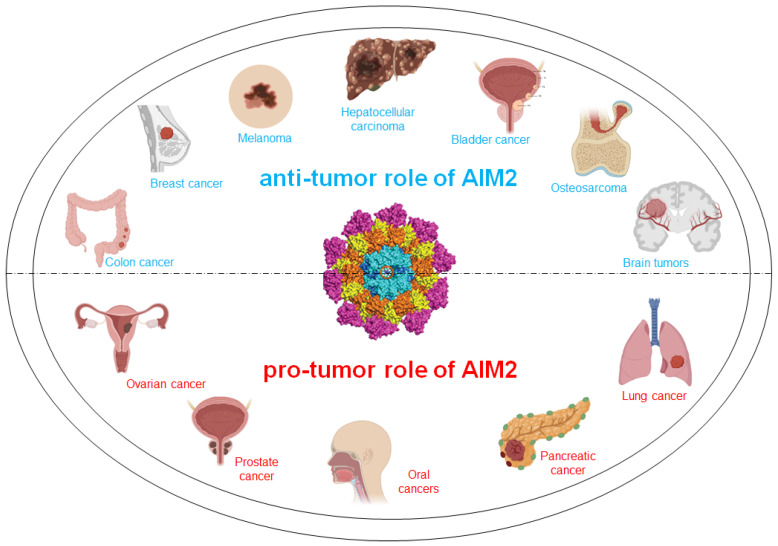
Role of AIM2 in distinct cancer types. AIM2 acts as a double-edged sword controlling cancer establishment and progression according to the biological diversity between different tumor types and the complex nature of cancer growth. Several pieces of evidence suggest that AIM2 plays an anti-tumor role in colon and breast cancer, melanoma, hepatocellular carcinoma, bladder cancer, osteosarcoma, and brain tumors. In contrast, AIM2 promotes the establishment and progression of ovarian and prostate cancer, oral malignancies, pancreatic and lung cancer.

The recognition of DNA/DNA-RNA hybrids by AIM2 leads to the assembly of the inflammasome complex characterized by caspase-1 (canonical pathway) or caspase-4/-8 (non-canonical pathway), with the ensuing release of active IL-1β and IL-18 [11,12,13] (Figure 1). Despite other sentinel receptors (NLRs, NOD-like receptors), AIM2 does not require the two-signal model, thus its expression is not dependent on the priming signal, as in the case of the NLR family pyrin domain containing 3 (NLRP3) [6]. It has to be noted, though, that dsDNA can also be sensed by the cGAS (cyclic GMP-AMP synthase)-STING (stimulator of interferon genes) signaling pathway [14], which has been reported as another cytoplasmic dsDNA sensor responsible for type I interferon (IFN) response [15] (Figure 1). Moreover, recently, it was suggested that dsDNA could induce AIM2 triggering in an inflammasome-independent manner through cGAS, which senses cytosolic DNA despite its length. Thus, despite the great interest in understanding the interplay between cGAS and AIM2-dependent pathways in cancer, this review will focus on AIM2, elucidating its dual role according to cancer type (Figure 2), taking into consideration the current therapeutic strategies for targeting the AIM2 inflammasome.

Initially, AIM2 was identified as a tumor suppressor in melanoma and colorectal cancer [16], but other studies on tumor-derived DNA [8,9], have demonstrated the pro-tumor activity of AIM2, as also demonstrated by our laboratory [12,17,18,19,20]. In support, the tumor secretome comprises DNA [9] that can either be detected by cells populating the tumor microenvironment (TME) or released into the blood as circulating-tumor DNA (ctDNA).

## 2. Anti-Tumor Role of AIM2 in Cancer

In the following subparagraphs, we summarize the studies that demonstrated that AIM2 has an anti-tumor function in colon, colorectal and breast cancer, melanoma, hepatocellular carcinoma, bladder cancer, osteosarcoma, and brain tumors (Figure 2).

### 2.1. Colon and Colorectal Cancer

Colon cancer is the third-most common type of cancer worldwide, accounting for more than 900,000 deaths per year [21]. It is well known that patients with inflammatory bowel disease, such as Crohn’s disease and ulcerative colitis, are more likely to be at risk of colorectal cancer (CRC), thus providing a link between inflammation and CRC [22].

Several pieces of scientific evidence have reported the ability of the AIM2 inflammasome to suppress colon cancer establishment and progression [23,24]. Patients with CRC are characterized by the genetic alteration/absence of AIM2 associated with an up to 3-fold increase in overall mortality and disease-specific mortality (HR = 3.14; 95% CI = 1.75–5.65) [23]. In support, Zhang et al. [25] found that AIM2 expression was lower in the primary CRC tissue than the adjacent normal tissue. Lower levels of AIM2 were correlated to both clinicopathological features, such as the depth of invasion, higher TNM clinical stage and lymph node metastasis, as well as to poor prognosis [25]. In support, a negative correlation between AIM2 expression and preoperative serum carcinoembryonic antigen (CEA) levels in CRC patients was reported [25]. These data suggest that a low expression of AIM2 could serve as an independent and significant prognostic factor for the poor survival of CRC patients. The potential value of the AIM2 gene as a biomarker of CRC prognosis was also suggested by Professor Tin’s group, who performed an extensive analysis of AIM2 and other NLRs in human CRC by combining the bioinformatics analysis of ten independent, publicly available, databases from the Oncomine^®^ Platform, an online collection of microarrays, with validation experiments using 40 case-matched cancer samples and adjacent healthy control tissues isolated from a cohort of patients from China [26]. The authors found that the AIM2 gene expression was diminished in CRC patients and its absence was correlated to cancer progression. Beyond its lower expression, the AIM2 gene contains a site for microsatellite instability that resulted in frequent gene mutations in CRC [27,28] and small bowel cancers [29]. The evaluation of mutations and the methylation of the AIM2 gene showed that it had a high percentage of single nucleotide variants (SNVs) and low methylation levels in colon cancer [30]. It has to be pointed out that, according to the well-known inverse correlation between gene expression and DNA methylation, the reduced methylation levels of the AIM2 gene [30] seems to be in contrast with the low AIM2 gene expression in colon cancer [23,25,26].

In support of the anti-tumor activity of AIM2 in CRC, the induction of AIM2 expression in HCT116 CRC cells by lentivirus transfection inhibited cell viability and increased the apoptosis rate. The inhibition of cell proliferation was associated with a diminished number of cells in the S phase, indicating that the expression of AIM2 could block the cell cycle transition from the G1 to S phase [24]. AIM2-induced apoptosis was mediated by the suppression of the phosphatidylinositol 3-kinase (PI3K)/protein kinase B (Akt) pathway. In support, in a mouse model of colitis-associated colon cancer (CAC) the genetic loss of AIM2 was associated with the activation of the DNA-dependent protein kinase (DNA-PK), a PI3K-related family member, which in turn promoted Akt phosphorylation and activation with an ensuing high tumor burden [24]. The pharmacological inhibition of Akt reduced the tumor load in AIM2 knockout mice, suggesting that Akt inhibitors could be used to treat AIM2-deficient human cancers.

Very recently, Pu et al. [31] demonstrated that low-dose chemotherapy induced cytosolic dsDNA accumulation and the ensuing activation of the AIM2 inflammasome [31]. The AIM2 activation was associated with an increased anti-tumor response after the anti-Programmed Cell Death 1 (PD-1) blockade, an effect that was attenuated in AIM2 knockout mice with colon cancer [31].

Collectively, these studies provide insights into the anti-tumor activity of AIM2 in colon cancer and CRC, paving the way for further investigation into therapeutic strategies targeting AIM2 in colon cancer and CRC patients (Figure 2).

### 2.2. Breast Cancer

Breast cancer (BC) was ranked as the first diagnosed cancer globally, causing 685,000 deaths in 2020, according to GLOBOCAN 2020 [32].

Recent evidence has highlighted that BC, which was historically defined as immunologically “cold” [33], is characterized by a heterogeneous and dynamic immune landscape, pointing at the importance of the TME in modulating the anti-tumor immune response [34]. In this context, several data reported the dual role of the inflammasome in BC, in that a pro- and anti-tumor activity of the NLRP3 and AIM2 inflammasomes was, respectively, suggested in this type of tumor [35]. Focusing on the AIM2 inflammasome, it was found that AIM2 expression suppressed the proliferation and tumorigenicity inducing tumor cells toward apoptosis [36]. As observed for colon cancer, the expression of AIM2 is low in BC samples according to The Cancer Genome Atlas (TCGA) and The Genotype-Tissue Expression (GTEx) databases [30]. Instead, a higher AIM2 expression score was correlated to a better prognosis of BC patients. In support of the anti-tumor function of AIM2 in BC, Chen et al. [36] demonstrated that the manipulation of the AIM2 gene inhibited mammary tumor growth in an orthotopic model of BC [36], paving the way for the possible application of gene therapy for BC patients based on overexpressing AIM2.

Recently, an interesting paper showed that the treatment with anti-PD1 antibodies led to antibody-dependent cellular phagocytosis (ADCP) which promoted the phagocytosis of the tumor cells and then the activation of AIM2 in macrophages due to the exposure of the tumor DNA. AIM2 activation led to the upregulation of programmed cell death ligand 1 (PD-L1) and Indoleamine 2, 3-dioxygenase (IDO) via IL-1β [37]. On the other hand, it was proved that cisplatin-based chemotherapy can increase the levels of PD-L1 [38], leading us to think that the combination of chemotherapy and immunotherapy could be effective in BC patients. Unfortunately, the treatment with Atezolizumab plus chemotherapy of triple-negative BC patients showed only a modest response and was associated with serious immune-mediated adverse events [39]. The latter results question the protective role of AIM2 in BC. In support, the blockade of IL-1β in a mouse model of BC reversed immunosuppression and completely abrogated tumor progression in combination with anti-PD-1 antibodies [40], suggesting a pro-tumor role of AIM2 in BC.

Therefore, further studies will be needed to clarify the role of the AIM2 inflammasome in breast cancer.

### 2.3. Melanoma

Melanoma is an aggressive skin cancer with high mortality, especially for patients at advanced stage [41]. The gene encoding AIM2 was originally isolated from human melanoma cells and identified as a gene in which expression was lost in these cells [42]. Early research has demonstrated that AIM2 overexpression is able to delay proliferation, increase cell death susceptibility, and reverse the melanoma phenotype [43]. Later, it was found that AIM2 was upregulated in acute and chronic inflammatory skin conditions [44], in melanocytic nevi and in most primary melanomas [45]. To note, a gene expression analysis in the public database of Skin Cutaneous Melanoma (SKCM) revealed that AIM2 was significantly down-expressed, that it had a high percentage of SNVs, and that its expression was negatively correlated with copy number variations (CNVs) [30], suggesting the anti-tumor role of AIM2 in melanoma.

In sharp contrast, Fukada et al. [4] found that AIM2 knockout mice had lower tumor growth than wild type mice challenged with melanoma B16-F10 cells [4]. Tumor-infiltrated dendritic cells (TIDCs) highly expressed AIM2 and its levels were associated with tumor progression in human melanoma patients [4]. Interestingly, the vaccination with AIM2-deficient DCs improved the efficacy of both adoptive T cell therapy and anti–PD-1 immunotherapy [4]. This aspect will be better illustrated in the following paragraph dedicated to the possible application of AIM2 inflammasome modulators.

Due to the discrepancies, it is clear that the role of AIM2 in melanoma requires further investigation.

### 2.4. Hepatocellular Carcinoma

Hepatocellular carcinoma (HCC) is the sixth-most commonly diagnosed cancer and the third leading cause of cancer death [46]. Cirrhosis and chronic hepatitis have been documented among the most common etiological factors for the development of HCC due to the establishment of an inflammation-based condition associated with fibrosis, necrosis, and regeneration, which contribute to tumorigenesis [47]. As already reported, the AIM2 inflammasome can also be activated by viral DNA [5,10]. Therefore, it is not surprising that AIM2 is involved in the immunopathology of virus infection-associated chronic hepatitis, as demonstrated by Han et al. [48], who found that AIM2 expression was elevated in patients affected by chronic hepatitis B [48]. According to these results and to the association between hepatitis and HCC development, it is likely for us to suppose the pro-carcinogenic role of AIM2 in liver tumorigenesis. However, it was revealed that the AIM2 inflammasome can suppress HCC in that its levels were significantly decreased in liver cancer tissues, and the loss of its expression was significantly correlated with more advanced tumor progression [49]. It was found that AIM2 overexpression inhibited the mammalian target of the rapamycin (mTOR)-ribosomal S6 kinase 1 (S6K1) pathway and further blocked proliferation, colony formation, and the invasion of HCC cells [49]. In addition, AIM2 overexpression inhibited HCC cell proliferation, migration, and invasion while promoting apoptosis and autophagy, effects that were blocked once AIM2 was knocked down [50]. To further support the anti-tumor role of AIM2 in HCC, it was recently found that a higher expression of AIM2 was associated with a better prognosis of HCC patients in terms of overall survival (OS), disease-specific survival (DSS), and progression-free interval (PFI) [30]. The latter data are in contrast with what was reported by a previously published paper which, instead, highlighted that AIM2 gene expression was lower in cancerous compared to non-cancerous tissues obtained from HCC patients [51]. In support, the genetic inactivation of AIM2 protected mice exposed to diethylnitrosamine (DEN) from HCC development [52], suggesting that AIM2 has a dichotomous role.

### 2.5. Bladder Cancer

Bladder cancer (BLCA) is the most common malignancy of the urinary system [53]. Most bladder cancers are diagnosed at an early stage, when the cancer is highly treatable; however, this tumor is associated with a high morbidity and mortality after chemotherapy. Although BLCA has been associated with inflammation, the precise molecular pathways involved in inflammation-related BLCA remain largely uncertain, although it was suggested that inflammatory cytokines (i.e., IL-1, IL-6, TNFα) could drive bladder carcinogenesis and suppress the anti-tumor immune response [54]. To our knowledge, only two studies have focused on the role of the AIM2 inflammasome in BLCA. In the first study, the expression of AIM2 was found to be lower in high-grade BLCA and correlated with a better prognosis of BLCA patients [55]. However, in a mouse model of BLCA overexpressing AIM2, tissue samples were more highly infiltrated by CD11b^+^ cells [55], suggesting that DNA fragments released by dead and tumor cells could lead to immune cell infiltration via AIM2. In the second study, which analyzed the gene expression of AIM2 in several cancer types, the authors found that AIM2 was more expressed in cancerous compared to non-cancerous tissues and that its higher expression was correlated to a good prognosis of patients affected by BLCA [30].

### 2.6. Osteosarcoma

Osteosarcoma, a malignant osseous neoplasm caused by osteoblastic mesenchymal cells, is the most common primary bone cancer in childhood [56]. Among the spectrum of molecular changes that occur in the pathological landscape of osteosarcoma, the oncogenic activation of mTOR signaling significantly contributes to osteosarcoma progression and metastasis [57]. Based on this concept and according to the proved link between the PI3K/AKT/mTOR signaling pathway and the AIM2 inflammasome in certain types of cancers [24,49], Zheng et al. [58] evaluated the possible crosstalk between the mTOR pathway and AIM2 inflammasome in osteosarcoma establishment [58]. The authors found that AIM2 expression was downregulated in osteosarcoma cells at both the transcriptional and protein level; instead, AIM2 overexpression led to the inhibition of proliferation, invasion, migration, and EMT, and induced apoptosis in osteosarcoma cells [58]. Interestingly, the levels of phosphorylated (p)-PI3K, p-AKT and p-mTOR were markedly downregulated following AIM2 overexpression. Similar effects were observed when the mTOR pathway was inhibited by means of LY294002.

To our knowledge, no other studies investigating the function and molecular mechanisms by which AIM2 is involved in osteosarcoma have been performed. Therefore, according to this unique study [58], the AIM2 inflammasome seems to play an anti-tumor role in osteosarcoma, most likely via the PI3K/AKT/mTOR signaling pathway.

### 2.7. Brain Tumors

Gliomas, accounting for 80% of primary malignant brain tumors, are classified into low- and high-grade gliomas according to the malignancy degree [59]. Low grade gliomas specifically represent 40% of all central nervous system tumors in children; instead, the majority of high-grade gliomas, also referred to as glioblastoma (GBM), occur de novo. GBM is the most common and lethal brain cancer in adults and one of the least immunogenic tumors, characterized by an immunosuppressed TME highly populated by tumor-infiltrated lymphocytes (TILs), myeloid-derived suppressor cells (MDSCs), and regulatory T cells (T regs) [60].

It is well known that high neuro-inflammation is pivotal in the genetic, molecular, and histological pathology of gliomas, and that the inflammatory microenvironment in the central nervous system has been closely linked to inflammasomes, which control the inflammatory response and coordinate innate host defense [61]. By using the public TCGA database, it was found that AIM2 was highly expressed in G2, G3, and G4 gliomas and was frequently altered in low grade gliomas [62]. The authors also highlighted that there was a significant inverse correlation between methylated CpG loci and AIM2 expression in glioblastoma patients. Moreover, the experimental data obtained by using multiple cell lines demonstrated that ASC and AIM2 protein expression was higher in lipopolysaccharide (LPS)-primed LN18 glioma and BV2 microglial cells compared to the control. However, this research did not rule out the anti- or pro-carcinogenic activity of AIM2 in the establishment and progression of gliomas and glioblastomas. Further studies by Chen et al. [63] revealed that the inhibition of the AIM2 inflammasome increased the proliferation of gliomas and increased temozolomide resistance in vitro, suggesting its anti-carcinogenic function [63]. To further support the anti-tumor role of AIM2 in brain cancers, very recently, it was demonstrated that Tumor Treating Fields (TTFields), an approved non-invasive regional anti-mitotic treatment for GBM, generated large cytosolic naked micronuclei clusters in GBM through the focal disruption of the nuclear envelope, thereby recruiting AIM2 and cGAS, leading to the release of pro-inflammatory cytokines and type I IFNs [64]. As previously mentioned, further explanations about the impact of AIM2 modulation as a promising approach for cancer therapy will be described in the following paragraphs.

Collectively, these studies highlighted the tumor suppressor function of AIM2 in brain cancers.

## 3. Pro-Tumor Role of AIM2 in Cancer

In the following subparagraphs, we reviewed the pro-tumor activity of AIM2, focusing on the recent findings about the involvement of the AIM2 receptor in the establishment and progression of ovarian and prostate cancer, oral malignancies, pancreatic and lung cancer (Figure 2).

### 3.1. Ovarian Cancer

Ovarian cancer is the leading cause of death in women, and it is associated with a 5-year survival of 15.7% at the local stage, and about 58% at the metastasized stage [65]. Several analyses revealed that inflammation and endometriosis play key roles in different stages of tumor development, including establishment, progression, malignant conversion, invasion, and metastasis [66]. A bioinformatic analysis revealed that among the dysregulated immune-/inflammation-related functions in ovarian cancer, the inflammasome complex activation is associated with malignant transformation and tumor progression [67]. The authors found that high expression levels of AIM2 mRNA were significantly correlated with poor progression-free survival (PFS), and that increased protein levels of AIM2 were associated with Ki67-positive staining in human tissues collected from ovarian cancer patients, supporting its role in cancer progression [67]. In support, AIM2 expression was significantly higher in patients who did not respond to bevacizumab therapy, associated with a worse PFS [68]. Although the precise mechanism by which AIM2 drives bevacizumab resistance still needs further investigation, these results reveal that AIM2 expression could be used as a histopathological biomarker to predict the therapeutic benefit of bevacizumab-based anti-angiogenic treatment in ovarian carcinoma patients. Moreover, a study carried out by Wang et al. [69] identified AIM2 as one of the genes associated with ovarian cancer patient prognosis by using a network-based method [69].

Collectively, these studies highlight that the AIM2 inflammasome might have a role in ovarian cancer establishment and progression and could be used as a valuable biomarker for patients’ prognosis. In this context, AIM2 could represent a potential therapeutic target for ovarian carcinoma treatment.

### 3.2. Prostate Cancer

Prostate cancer is the second-most frequent malignancy in men and the fifth leading cause of death worldwide [70]. Many prostate cancers are detected on the basis of elevated plasmatic levels of the prostate-specific antigen (PSA), a glycoprotein normally expressed by prostate tissue, whose levels are often increased in intraprostatic inflammation [71]. In this context, it was reported that inflammation is associated with high-grade or aggressive prostate tumors and metastatic spread, suggesting the strong link between prostate and inflammation [72]. A recent study has shown that the AIM2 inflammasome plays a critical role in both human prostatic diseases, such as benign prostate hyperplasia (BPH) and prostate cancer [73]. It was found that AIM2 expression was induced in human prostate epithelial cells by IFNα, IFNβ and IFNγ and its activation was triggered by cytosolic DNA. The authors also found that the basal levels of AIM2 mRNA were significantly lower in clinical tumor specimens, but higher in BPH, compared to normal prostate tissue [73]. This led us to think that AIM2 overexpression was mainly observed during prostatic infection, but the activation of the Nuclear factor kappa-light-chain-enhancer of activated B cells (NF-kB) during hypoxic conditions, typical of prostate cancer, can increase AIM2 expression [74], a result confirmed by Qin et al. [30], who found that the AIM2 gene was expressed at high levels in prostatic adenocarcinoma with a dismal prognosis [30].

According to these pieces of evidence, it is possible to conclude that the AIM2 inflammasome has a pro-tumor function in prostate cancer.

### 3.3. Oral Cancers: Head and Neck Squamous Cell Carcinoma, Oral Squamous Cell Carcinoma and Hypopharyngeal Squamous Cell Carcinoma

Oral cancers are among the most common malignancies worldwide. Head and neck squamous cell carcinoma (HNSCC) is the sixth-most common malignancy [75]. Hypopharyngeal squamous cell carcinoma (HSCC) is one of the subtypes of HNSCC, accounting for around 2–6% of HNSCC cases [76]. Oral squamous cell carcinoma (OSCC) represents the most common type of head and neck malignancy, according to the data collected by the Global Cancer Observatory (GCO) (World Health Organization—Global Cancer Observatory-GLOBOCAN, 2020) [46].

Although smoking and alcohol are the main causes of HNSCC, recent evidence has highlighted that the human papillomavirus (HPV) infection represents a risk factor for the establishment of this tumor [77]. Thus, according to the role of the innate immune system in providing non-specific protection and enhancing the adaptive immune response against a variety of pathogens, including HPV, a role for the cytosolic dsDNA sensor AIM2 in oral cancer was suggested. Riva et al. [78] have recently investigated the gene expression of AIM2 in 34 HNSCC patients undergoing surgical treatment and the possible correlation between AIM2 levels and HPV infection status, clinical characteristics, and survival [78]. AIM2 gene overexpression was mainly 10% versus 50% in HPV^+^ HNSCCs versus HPV^−^ tumors, respectively [78]. In addition, a bioinformatic analysis revealed that the elevated AIM2 expression in HNSCC was positively correlated with disease stage and HPV infection, thereby possessing both diagnostic and prognostic significance [79]. Ectopic AIM2 expression promoted cell growth, migration, tumorigenesis, and metastasis both in vitro and in vivo through the IL-17-MAPK signaling pathway [79].

In 2012, Kondo et al. [80] found that knocking down AIM2 in OSCC cells, which harbor reduced p53 activity, resulted in the suppression of cell growth and apoptosis, accompanied by the downregulation of NF-κB activation, an event that was not observed in p53 wild-type cell lines [80]. These data highlighted, for the first time, that the expression of AIM2 may have oncogenic activities in OSCC cells characterized by the inactivation of p53. Later, the same research group investigated the possible contribution of AIM2 in the progression of OSCC metastasis [81]. They found that the overexpression of AIM2 was correlated to the increased migration and invasion capacity of OSCC cells and enhanced EMT in vitro, higher tumor growth in the tongue, and decreased survival in vivo, highlighting that AIM2 in OSCC promotes cancer growth and progression [81].

In another study, low levels of AIM2 combined with a high expression of phosphorylated-Signal transducer and activator of transcription 3 (p-STAT3) were correlated to a worse prognosis of HSCC patients [82]. Particularly, the authors found that although AIM2 expression was higher in adjacent normal hypopharyngeal tissues than HSCC tissues, lower expression was positively correlated with lymph node metastasis and intravascular tumor thrombus, implying the poor prognosis of patients with HSCC [82].

All together, these pieces of evidence suggest the pro-carcinogenic role of AIM2 in oral cancers.

### 3.4. Pancreatic Cancer

Pancreatic cancer is one of the most aggressive and lethal cancers, with insidious onset and poor prognosis [83]. According to GLOBOCAN 2020, pancreatic cancer is the twelfth most common malignancy and the seventh leading cause of cancer mortality [46]. The major genetic changes in carcinogenesis of the pancreas include the activation of the Kirsten rat sarcoma virus (KRAS) and the inactivation of tumor protein p53 (TP53), Cyclin Dependent Kinase Inhibitor 2A (CDKN2A), and SMAD Family Member 4 (SMAD4) [84]. Among the risk factors for the establishment of pancreatic cancer, a key role is played by uncontrolled pancreas inflammation which paves the way for chronic pancreatitis and the alteration of endocrine and exocrine pancreas functions [84]. A previous study has already highlighted that circulating immune cells isolated by patients with acute pancreatitis were characterized by the increased expression and activation of AIM2 during the onset of the disease [85]; moreover, AIM2 levels and the AIM2-associated IL-1β release by peripheral blood mononuclear cells (PBMCs) were correlated to systemic inflammation in these patients [85]. Li et al. [86], by taking advantage of pink1 knockout- and park2 knockout-mice found that mitochondrial iron-mediated oxidative DNA injury led to AIM2 inflammasome activation in pancreatic ductal adenocarcinoma (PDAC) cells, which in turn induces High Mobility Group Box 1 (HMGB1) release and the subsequent CD274 (encoding PD-L1) expression [86]. Interestingly, the genetic ablation of AIM2 resulted in a reduced pancreatic neoplasia burden [86]. These data demonstrated both the oncogenic function of the AIM2 inflammasome in pancreatic carcinogenesis and the role of the mitochondrial-targeted protein Phosphatase and tensin homolog (PTEN)-induced putative kinase 1 (PINK1) and PRKN/ parkin RBR E3 ubiquitin protein ligase (PARK2) system on the oncogenic KRAS-driven PDAC.

### 3.5. Lung Cancer

Lung cancer is the most common solid tumor, accounting for an estimated 1.8 million of cases in 2020 and the leading cause of cancer-related deaths worldwide [87]. Non-small cell lung cancer (NSCLC) is the most frequent type of lung cancer accounting for about 85% of cases and it is mainly associated with cigarette smoke and air pollution exposure. The impact of chronic inflammation in lung cancer is described and proved by the detection of pro-inflammatory cytokines and immune infiltrates in samples obtained by lung cancer patients, such as the detection of IL-1 like cytokines, whose expression is correlated to the activation of the inflammasome complex, in both plasma and tissues samples of lung cancer patients [88]. In our previous studies, we demonstrated that the AIM2 inflammasome and its related cytokines participate in lung tumor growth in NSCLC human samples [15]. We observed that lung tumor masses were highly populated by plasmacytoid dendritic cells (pDCs) in their immunosuppressive phenotype. Particularly, tolerogenic cancerous pDCs were able to produce high levels of IL-1α after the stimulation of the AIM2 inflammasome, which, in turn, was responsible for calcium efflux and reactive oxygen species (ROS) from mitochondria, leading to calpain activation facilitating tumor cell proliferation in the lung. Recently, we demonstrated that AIM2 was expressed in lung tumor tissues according to the TNM stage and an immunosuppressive signature [20]. The analysis of the bulk RNA sequencing (RNA-seq) database from lung adenocarcinoma (LUAD) patients demonstrated that, according to mRNA expression, AIM2-positive LUAD tissues were characterized by the increased recruitment of resting myeloid DCs (mDCs) and CD4-positive memory T cells that collaborated to create an immunosuppressive TME facilitating tumor growth at the basis of the dismal prognosis of patients [19]. Interestingly, we also demonstrated that a worse survival rate related to the expression of AIM2 mRNA was correlated to an inflammatory profile characterized by inflammasome-dependent IL-1-like cytokines in non-smoker and smoker LUAD patients, but not in LUAD patients who quit smoking. In support of our data, other research groups proved the oncogenic role of AIM2 in NSCLC in both in vitro and in vivo experiments [89]. Interestingly, Qi et al. [90] proved the colocalization of AIM2 with mitochondria in NSCLC cells and that the loss of AIM2 induced the increase in mitochondrial fusion through mitofusin 2 (MFN2) upregulation and the decrease in cell proliferation [90].

It is well known that the AIM2 inflammasome is involved in the inflammatory pattern associated with chronic pulmonary diseases, such idiopathic pulmonary fibrosis (IPF) [91], long-Coronavirus Disease 19 (COVID-19) [92] and chronic obstructive pulmonary disease (COPD) [93]. The latter is an inflammatory pulmonary disorder associated with a progressive and irreversible decline in lung function, which is considered as another risk factor for NSCLC establishment. COPD and lung cancer share common etiological insults (tobacco and environmental pollutant exposure) and lung-associated chronic inflammation [94]. Our research group found that AIM2 is highly expressed in circulating cells collected by exacerbated COPD patients and that its stimulation via Poly dA:dT induced the release of IL-1α in a canonical, caspase-1-dependent, and non-canonical, caspase-4-dependent manner, which in turn, was responsible for the release of the immunosuppressive and pro-fibrotic cytokine TGF-β [13,95]. Moreover, using a mouse model of cigarette smoking-induced COPD, we demonstrated that smoking exposure led to the release of inflammasome-associated IL-1-like cytokines (IL-1α, IL-1β, IL-33, IL-18) and increased the expression of AIM2 in lung-recruited macrophages in wild type C57Bl/6 mice, but not in 129Sv mice, who lack a functional caspase-11, the murine analog of human caspase-4 [96,97,98]. Thus, AIM2 was identified as a novel diagnostic tool to predict lung cancer establishment [18,19,20].

In accordance with the previous data about the pro-carcinogenic role of AIM2 in lung cancer, Zheng et al. [99] demonstrated that the upregulation of mRNA levels of AIM2 in tumor tissues was associated with the absence of Epidermal growth factor receptor (EGFR)/KRAS/anaplastic lymphoma kinase (ALK) mutations [99]. In vitro experiments proved that AIM2 knockdown blocked the migration ability and lung colony-forming ability of LUAD cells; meanwhile, the overexpression of AIM2 was correlated to enhanced PD-L1 expression in LUAD cells [99]. In addition to this latter research, a very recent study has defined an inflammasome-independent role of AIM2 in KRAS-addicted LUAD [100]. The authors found that the genetic ablation of AIM2 in mutated Kras^G12D^ LUAD mouse models significantly reduced tumor growth, and that AIM2 expression was required in both hematopoietic and non-hematopoietic cellular compartments for Kras G12D-driven LUAD establishment [100].

All together, these studies provide insights into the pro-tumor function of AIM2 in lung cancer, paving the way for further studies focusing on therapeutic strategies targeting AIM2 in order to counter lung carcinogenesis progression.

## 4. Targeting AIM2 Inflammasome as a Therapeutic Approach in Cancer

The relevance of the AIM2 inflammasome in several types of tumors suggests the potential benefit of its therapeutic modulation in a range of cancers. Here, we reported the recently developed therapeutic approaches targeting the AIM2 inflammasome to affect cancer development. However, the utility of these therapeutic strategies will vary depending on the function of AIM2 in different tumors.

**Figure 3:**
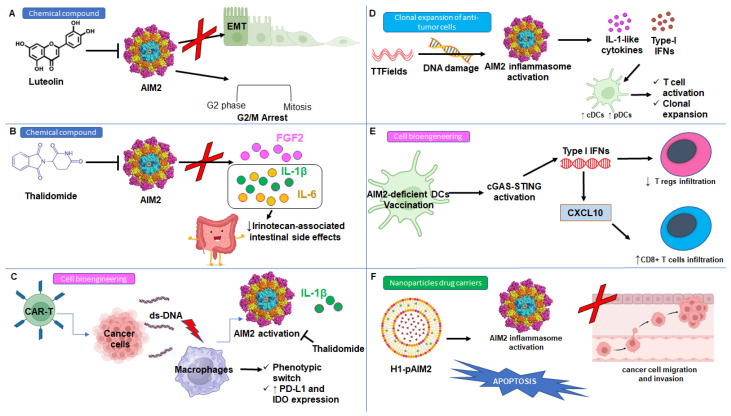
Schematic diagram of the therapeutic strategies targeting the AIM2 inflammasome in cancer. (**A**) The natural flavonoid luteolin exerts an anti-tumoral activity by blocking the AIM2 inflammasome activation leading to the G2/M phase arrest of the cell cycle and the inhibition of the epithelial to mesenchymal transition (EMT). (**B**) The AIM2 inflammasome inhibition by means of Thalidomide induces a lower release of Fibroblast growth factor 2 (FGF2) and IL-1β and IL-6, reducing the tumor growth associated with reduced pro-inflammatory cytokines ameliorating the intestinal side effects associated with irinotecan treatment in cancer patients. (**C**) Chimeric antigen receptor T (CAR-T) treatment induced AIM2 inflammasome activation in macrophages with the ensuing release of IL-1β and macrophages phenotypic switch, which highly expressed programmed cell death ligand 1 (PD-L1) and indoleamine 2,3-dioxygenase (IDO). (**D**) Tumor treating fields (TTFields) led to AIM2 triggering, favoring the activation of adaptive immunity via IL-1-like cytokines and a type I IFNs release leading to DC activation and favoring T cell activation and clonal expansion. (**E**) The vaccination with AIM2-deficient DCs promotes the activation of the cGAS-STING pathway, which is responsible for the release of type I IFN secretion which favors the infiltration of CD8+ T cells through the production of CXCL10 and reduces the recruitment of regulatory T cells (T regs). (**F**) The use of nanoparticle drug carriers for plasmid delivery, the Folate-grafted PEI600-CyD (H1)-based plasmid AIM2 (pAIM2) nanoparticles, is able to augment the expression of AIM2, to trigger cells apoptosis, and to limit cancer cell migration and invasion.

Very recently, luteolin, a natural flavonoid well known for its anti-inflammatory properties [101] was reported as being able to mediate the anti-tumor effect in NSCLC in an AIM2-dependent manner [102]. The authors highlighted that luteolin blocked AIM2 inflammasome activation leading to the G2/M phase arrest, inhibiting EMT in vitro and in vivo (Figure 3A). Interestingly, these effects were abolished by silencing AIM2, but not by AIM2 overexpression [102], indicating that strategies to downregulate AIM2 might be an effective way for NSCLC treatment.

Among the available options to attenuate dsDNA-mediated inflammasome activation, the immunomodulatory drug thalidomide was proved to be able to inhibit both caspase-1 [103] and AIM2 inflammasome [104]. In 2007, the Food and Drug Administration (FDA) approved thalidomide in association with dexamethasone for malignant treatment [105] in that it exerted strong anti-inflammatory and anti-angiogenetic activities by limiting the release of the pro-angiogenic growth factor Fibroblast growth factor 2 (FGF2) and pro-inflammatory cytokines IL-1β and IL-6 [103] (Figure 3B). The pharmacological inhibition of AIM2 by means of thalidomide significantly limited the intestinal toxicity associated with irinotecan without affecting its anticancer efficacy [104] (Figure 3C). Moreover, the addition of thalidomide to docetaxel seems to be a promising therapeutic approach to metastatic androgen-independent prostate cancer (AIPC), as proved by the results of a randomized phase II trial showing lower PSA levels and an improved median survival rate in patients treated with the combination regimen (Table 1) [106].

In a recent study it was found that Chimeric Antigen Receptor T cell (CAR-T) treatment led to the activation of AIM2 in macrophages and the ensuing release of IL-1β both by inducing the α1- adrenergic receptor (α1-AR)-mediated adrenergic signaling and by releasing tumor cell DNA [107]. CAR-T treatment induced a phenotypic switch of the macrophages which highly expressed immunosuppressive biomarkers as PD-L1 and IDO that, together with the release of IL-1β, could be responsible for the side effects of CAR-T therapy [108] (Figure 3C). Interestingly, the authors demonstrated that the combination of CAR-T treatment with a drug able to inhibit the AIM2 inflammasome (i.e., thalidomide) or to block of the α1-AR reversed the upregulation of PD-L1 and IDO and the phenotypic switch of the macrophages, limiting CAR-T therapy-associated toxic side effects and ensuring its anti-tumor effects (Table 1) [107]. Based on these results, it is possible to speculate that the combination of AIM2 inflammasome modulators, as in the case of thalidomide, with conventional chemotherapeutic drugs or immunotherapies could improve the anti-tumor effects in patients with advanced cancer, including those who develop chemotherapy resistance or undergo metastatic disease. At the same time, it was recently demonstrated that Doxorubicin induced the activation of the AIM2 and NLRP3 inflammasomes in both macrophages and neutrophils, leading to inflammatory cell death pyroptosis and the formation of neutrophil extracellular traps (NETs), which were responsible for the bone-damaging effects of this chemotherapeutic agent (Table 1) [109]. The deletion of AIM2 and/or NLRP3 mitigated the bone loss caused by doxorubicin, shedding lights on the potential association of AIM2 inflammasome inhibitors with adjuvant therapy to limit the risk of osteoporosis and fractures in cancer patients treated with chemotherapy.

Very recently, Green et al. [110] demonstrated that 4-sulfonic calixarenes are potent inhibitors of dsDNA-driven inflammation through the AIM2 inflammasome by means of both in vitro, in vivo, and in silico approaches [110]. The authors found that 4-sulfonic calixarenes were able to reversibly bind to the dsDNA-binding site in the HIN domain of AIM2, preventing inflammasome assembly, although at higher doses, these compounds also abrogated the activity of other two dsDNA sensors, cGAS and Tool-like receptor 9 (TLR9) [110]. Similar results were found for the polyanionic drug suramin, previously described as a purinergic receptor antagonist [111], which, due to the presence of multiple sulfonic acid groups, was identified as a compound with comparable properties to 4-sulfonic calixarenes [110]. It has to be pointed out that the ability of 4-sulfonic calixarenes to block AIM2 activity was evaluated in a model of post-stroke immunosuppression and T cell death. Nevertheless, based on the detrimental effect of AIM2 activation in the establishment and progression of certain types of cancer, and on the proved anticancer activity of calixarene-based compounds [112], it is possible to hypothesize the use of 4-sulfonic calixarenes as an effective inhibitor of the AIM2 inflammasome, pointing at future translational opportunities to target AIM2 in cancer patients.

As previously reported, the anti-tumor role of AIM2 has been reported in brain cancer, including gliomas [62]. In this context, it was observed that the treatment with TTFields led to both AIM2 assembly and activation (Figure 3D) (Table 1) and the induction of the cGAS-STING pathway, both responsible for the membrane-damaged cell death of glioblastoma cancer stem-like cells derived by patients [64]. The treatment with TTFields led to adaptive immunity activation in glioblastoma patients via a type I IFNs-based trajectory that reversed the local and systemic immunosuppression through cytotoxic lymphocyte activation, clonal expansion, and immune checkpoint upregulation [64]. To note, TTFields therapy increases the number of pDCs and induces the activation of both pDCs and conventional DCs (cDCs) favoring T cell activation and clonal expansion (Figure 3D). Focusing on the DC compartment, it is well known that the recruitment of AIM2-expressing pDCs and resting DCs in NSCLC is associated with the establishment of immunosuppressive TME [17,19], as also observed in melanoma [4]. Interestingly, as mentioned above, it was highlighted that vaccination with AIM2-deficient DCs ameliorated adoptive T cell therapy and anti–PD-1 immunotherapy efficacy by promoting STING-induced type I IFN secretion, favoring CD8+ T cell infiltration through the production of CXCL10 while limiting the accumulation of T regs and the production of IL-1β and IL-18 production in response to tumor-derived DNA (Figure 3E) [4]. These results reveal that silencing AIM2 in DC vaccination could be used as an alternative therapeutic approach to improve the efficacy of immunotherapy in melanoma patients and achieve therapeutic efficacy even for patients with cold tumors.

Over the past few years, the intensive research and progress in the field of nanotechnology-based therapies has boosted the development of nanoparticle drug carriers for nucleic acid delivery, including small interfering RNA (siRNA), mRNA, DNA, and oligonucleotides, to create therapeutics that can modulate protein deregulation [113]. In this context, Chai et al. [114] evaluated the therapeutic efficacy of Folate-grafted PEI600-CyD (H1)-based plasmid AIM2 (pAIM2) in renal cancer carcinoma (RCC), a type of tumor where AIM2 expression was significantly decreased. The authors found that H1/pAIM2 nanoparticles increased AIM2 expression and cell apoptosis, while decreasing cell migration and invasion in vitro (Figure 3F); moreover, in a 786-O-xenograft model, the intra-tumor administration of H1/pAIM2 nanoparticles reduced tumor growth [114]. According to these findings, the modulation of AIM2 inflammasome activation by means of H1/pAIM2 nanoparticles might represent a novel therapeutic strategy for RCC pathogenesis (Table 1).

**Table 1:** Research-based data on AIM2 blockade.

Therapeutic Agent/s	Experimental Approach	Results	References
AIM2-deficient DC vaccine plus adoptive T cell therapy and anti–PD-1 immunotherapy	In vivo and ex vivo study	Silencing AIM2 in DC vaccination improves the efficacy of immunotherapy in melanoma	[4]
Docetaxel plus Thalidomide	Randomized phase II clinical trial	The addition of thalidomide to docetaxel resulted in an encouraging PSA decline rate and overall median survival rate in patients with metastatic AIPC	[106]
CAR-T and Thalidomide	In vitro study	CAR-T therapy combined with Thalidomide limits IL-1β-related toxic side effects of CAR-T treatment and improves the anti-tumor effect of CAR-T therapy.	[107]
Doxorubicin	In vivo and ex vivo study	AIM2 inflammasome signaling insufficiency (in AIM2 knock-out mice) limits Doxorubicin bone-damaging effects	[109]

Abbreviations: DC—dendritic cells; PD-1—programmed cell death receptor 1; PSA—prostate-specific antigen; AIPC—androgen-independent prostate cancer; CAR-T—Chimeric Antigen Receptor T.

Beyond the AIM2-based nanotechnology therapeutic approach, genomic editing tools, such as Clustered Regularly Interspaced Short Palindromic Repeats (CRISPR)-associated (Cas) nuclease 9 (CRISPR-Cas9), could represent an optimal option for directly targeting AIM2 inflammasome. As anticipated above, it was observed that the deletion of the AIM2 gene using the CRISPR-Cas9 system significantly reduced tumor cell proliferation and temozolomide resistance in vitro [63]. Although these results pave the way to the application of CRISPR-Cas9 technology to manipulate the AIM2 inflammasome pathway, to date, no experimental data have been obtained by primary cancer cells and/or cancer mouse models to translate them into clinical practice.

Another aspect of the AIM2 inflammasome which needs attention is that it could be activated by radiotherapy [115], through which its anti-tumor effects are based on direct DNA damage through radiation and indirect DNA damage through ROS, resulting in apoptosis [116]. According to the concept that ionizing radiation-induced AIM2 inflammasome activation has been associated with the worsening of post-radiation side effects [115], the pharmacological modulation of AIM2 inflammasome activity could emerge as a potential avenue for reducing the negative effects of radiotherapy on patients. Thus, it was observed that Andrographolide, an active component extracted from Andrographis paniculate, significantly inhibited AIM2 inflammasome mediated-pyroptosis in macrophages by abrogating AIM2 from translocating into the nucleus to sense DNA damage induced by radiation or chemotherapeutic agents [117]. Specifically, the authors demonstrated that radiation exposure led AIM2 toward the sites of dsDNA breaks co-localizing with ASC, and the event abrogated after the treatment with Andrographolide [117]. More interestingly, recent evidence has highlighted that AIM2 could improve the anti-tumor effects of radiotherapy by triggering IL-1 signaling in DCs, leading to their activation and enhancement of T cell immunity [118]. In sharp contrast, Chiu et al. [119] found that AIM2 is a critical player for promoting radio-resistance and metastasis in refractory OSCC [119]. The authors showed that a higher expression of AIM2 in tumor tissues was correlated to the poor prognosis of patients and that AIM2 upregulation reinforced the transcriptional regulatory activity of STAT1/NF-κB towards the PD-L1 gene. In contrast, it was observed that increased AIM2 levels highly correlated with a favorable responsiveness to ICIs [119].

## 5. Conclusions

Besides the extensive studies on the involvement of the AIM2 inflammasome in innate immunity [5,6], the evidence summarized in this review highlight its implication in several malignancies. Considering the complex nature of tumor growth and the obvious biological diversity between cancer types, it is not surprising that the AIM2 inflammasome could exert both a tumor-promoting or a tumor-suppressive role. Therefore, according to the cancer type, it is very important to understand whether AIM2 could represent a pharmacological tool in cancer. In our opinion, a better understanding of both the activators and silencers of inflammasomes in the TME during tumor progression (i.e., tumor cell-derived soluble factors and other molecules like lactate or ATP released by dying tumor cells) and of the intricate crosstalk between tumor and immune cells is necessary in order to define if anti-AIM2-based drugs can find therapeutic applicability.
