# Correction: Colarusso et al. Role of the AIM2 Inflammasome in Cancer: Potential Therapeutic Strategies. *Biomedicines* 2025, *13*, 395

**DOI:** 10.3390/biomedicines13122895

**Published:** 2025-11-27

**Authors:** Chiara Colarusso, Michela Terlizzi, Simone Di Caprio, Anna Falanga, Emmanuel D’Andria, Roberta d’Emmanuele di Villa Bianca, Rosalinda Sorrentino

**Affiliations:** 1 Department of Pharmacy (DIFARMA), University of Salerno, 84084 Fisciano, SA, Italy; ccolarusso@unisa.it (C.C.); mterlizzi@unisa.it (M.T.); sdicaprio@unisa.it (S.D.C.); afalanga@unisa.it (A.F.); emmdandria@unisa.it (E.D.); 2 Department of Pharmacy, University of Naples Federico II, 80131 Naples, NA, Italy; roberta.demmanueledivillabianca@unina.it

**Text Correction**

In the original publication [1], as ref. 50 was retracted, it has been replaced with another reference. In this case, the main text has been revised accordingly.

A correction has been made to Section 2, Section 2.4, Paragraph 1:

Original text:

“In an in vitro study, knocking down AIM2 in HCC cells enhanced cell migration, the formation of cell pseudopodium, wound healing, and led to the activation of epithelial–mesenchymal transition (EMT) tumor metastasis, whereas the reintroduction of AIM2 attenuated these effects [50].”

Revised text:

“In addition, AIM2 overexpression inhibited HCC cell proliferation, migration, and invasion while promoting apoptosis and autophagy, effects that were blocked once AIM2 was knocked down [50].”

**Reference**

As ref. 50 was retracted, it has been replaced with another reference.

Original reference:

“50. Chen, S.L.; Liu, L.L.; Lu, S.X.; Luo, R.Z.; Wang, C.H.; Wang, H.; Cai, S.H.; Yang, X.; Xie, D.; Zhang, C.Z.; et al. HBx-mediated decrease of AIM2 contributes to hepatocellular carcinoma metastasis. *Mol. Oncol.* **2017**, *11*, 1225–1240”.

Revised reference:

“50. Xie, S.; Wang, C.; Liu, X.; Li, C.; Yu, J.; Ma, S.; Li, Q.; Du, W. Hepatocellular carcinoma and AIM2: Therapeutic potential through regulation of autophagy and macrophage polarization. *Immun. Inflamm. Dis.* **2024**, *12*, e70002”.

The authors state that the scientific conclusions are unaffected. This correction was approved by the Academic Editor. The original publication has also been updated.
